# Unique Features of Cardiovascular Involvement and Progression in Children with Marfan Syndrome Justify Dedicated Multidisciplinary Care

**DOI:** 10.3390/jcdd11040114

**Published:** 2024-04-03

**Authors:** Anwar Baban, Giovanni Parlapiano, Marianna Cicenia, Michela Armando, Alessio Franceschini, Concettina Pacifico, Arianna Panfili, Gaetano Zinzanella, Antonino Romanzo, Adelaide Fusco, Martina Caiazza, Gianluigi Perri, Lorenzo Galletti, Maria Cristina Digilio, Paola Sabrina Buonuomo, Andrea Bartuli, Antonio Novelli, Massimiliano Raponi, Giuseppe Limongelli

**Affiliations:** 1 The European Reference Network for Rare, Low Prevalence and Complex Diseases of the Heart-ERN GUARD-Heart, Cardiogenetic Center, Bambino Gesù Children’s Hospital, Istituto di Ricovero e Cura a Carattere Scientifico (IRCCS), 00165 Rome, Italy; giovanni.parlapiano@opbg.net (G.P.); ari.panfili24@gmail.com (A.P.); 2 The European Reference Network for Rare, Low Prevalence and Complex Diseases of the Heart-ERN GUARD-Heart, Pediatric Cardiology and Arrhythmia/Syncope Units, Bambino Gesù Children’s Hospital, IRCCS, 00165 Rome, Italy; marianna.cicenia@opbg.net (M.C.); alesssio.franceschini@opbg.net (A.F.); 3 Department of Neuroscience and Neurorehabilitation, Bambino Gesù Children’s Hospital, IRCCS, 00168 Rome, Italy; michela.armando@opbg.net; 4 Audiology and Otosurgery Unit, Bambino Gesù Children’s Hospital, IRCCS, 00165 Rome, Italy; concettina.pacifico@opbg.net; 5 Ophthalmology Unit, Bambino Gesù Children’s Hospital, IRCCS, 00165 Rome, Italy; gaetano.zinzanella@opbg.net (G.Z.); antonino.romanzo@opbg.net (A.R.); 6 Inherited and Rare Cardiovascular Diseases, Department of Translational Medical Sciences, University of Campania “Luigi Vanvitelli”, Monaldi Hospital, 80131 Naples, Italy; adelaide.fusco@ospedalideicolli.it (A.F.); martinacaiazza@libero.it (M.C.); limongelli.giuseppe@libero.it (G.L.); 7 Department of Pediatric Cardiology and Cardiac Surgery, Heart and Lung Transplant, Bambino Gesù Children’s Hospital, IRCCS, 00165 Rome, Italy; gianluigi.perri@opbg.net (G.P.); lorenzo.galletti@opbg.net (L.G.); 8 Genetics and Rare Diseases Research Division, Bambino Gesù Children’s Hospital, IRCCS, 00165 Rome, Italy; mcristina.digilio@opbg.net (M.C.D.); psabrina.buonuomo@opbg.net (P.S.B.); andrea.bartuli@opbg.net (A.B.); 9 Translational Cytogenomics Research Unit, Laboratory of Medical Genetics, Bambino Gesù Children’s Hospital, IRCCS, 00146 Rome, Italy; antonio.novelli@opbg.net; 10 Medical Direction, Bambino Gesù Children’s Hospital, IRCCS, 00165 Rome, Italy; massimiliano.raponi@opbg.net; 11 Centre for Paediatric Inherited and Rare Cardiovascular Disease, Institute of Cardiovascular Science, University College London, London WC1N 3JH, UK

**Keywords:** Marfan Syndrome, children, multidisciplinary management, variability, multisystemic, personalized approach, age related penetrance

## Abstract

Marfan syndrome (MIM: # 154700; MFS) is an autosomal dominant disease representing the most common form of heritable connective tissue disorder. The condition presents variable multiorgan expression, typically involving a triad of cardiovascular, eye, and skeletal manifestations. Other multisystemic features are often underdiagnosed. Moreover, the disease is characterized by age related penetrance. Diagnosis and management of MFS in the adult population are well-described in literature. Few studies are focused on MFS in the pediatric population, making the clinical approach (cardiac and multiorgan) to these cases challenging both in terms of diagnosis and serial follow-up. In this review, we provide an overview of MFS manifestations in children, with extensive revision of major organ involvement (cardiovascular ocular and skeletal). We attempt to shed light on minor aspects of MFS that can have a significant progressive impact on the health of affected children. MFS is an example of a syndrome where an early personalized approach to address a dynamic, genetically determined condition can make a difference in outcome. Applying an early multidisciplinary clinical approach to MFS cases can prevent acute and chronic complications, offer tailored management, and improve the quality of life of patients.

## 1. Introduction

Marfan Syndrome (MIM: # 154700; MFS) is an autosomal dominant (AD) multisystemic condition typically characterized by cardiovascular, ocular, and musculoskeletal system involvement. MFS represents the “paradigm disease” of connective tissue disorders (CTDs); in fact, any organ or system with connective components can be affected. The estimated prevalence of MFS indicates that it occurs in between 1 in 5000 and 1 in 10,000 people (Dietz GeneReviews, 2001–2022) [1]. This might be underestimated, as major organ involvement is occasionally absent, making diagnosis more difficult.

MFS has age-related penetrance. In other words, major and minor features can be progressive in nature, and an extreme variability in disease expression is possible. On one hand, the diagnostic approach to MFS is certainly more easily applied in adult patients, considering that the phenotypic characteristics and clinical features of the condition are more evident and expressed in this population. On the other hand, confirming a clinical suspicion of MFS in children can be more complex and not definitive due to the age-related penetrance of the disorder and the possibility that certain manifestations may only be presented later in life (often after the age of 20) (Lodato et al., 2022) [2].

Moreover, in children, the diagnosis can be secondary to family member screening after the identification of a molecularly proven parent with *FBN1* (likely, L.) pathogenic variants. In cases of children with a positive genotype but a negative phenotype, it is necessary to share knowledge with parents regarding potential age-related penetrance of the condition, and to schedule personalized surveillance of the main “target organs” in MFS.

From the genetic point of view, MFS is caused by *FBN1* (L.) pathogenic variants (OMIM* 134797), located on the long arm of chromosome 15 (15q21). This gene is relatively large, and the coding sequence consists of 65 exons (Corson et al., 1993) [3].

In individuals with classical MFS, the detection rate using genetic analysis is high, reaching approximately 95% of cases. In 90–93% of cases, an *FBN1* (L.) pathogenic variant is identifiable through Next Generation Sequencing (NGS) analysis. Five percent of elusively diagnosed patients carry copy number variants (FBN1 deletions or duplications) that can be disclosed through multiplex ligation-dependent probe amplification (MLPA) or chromosomal microarrays (Dietz GeneReviews, 2001–2022) [1].

An important step in the NGS molecular diagnosis of MFS through FBN1 analysis encompasses DNA variants classification. According to the American College of Medical Genetics and Genomics Guidelines (ACMG), these are divided into five classes: benign variant (Class 1), likely benign variant (Class 2), variant of uncertain significance (VUS) (Class 3), likely pathogenic variant (Class 4), and pathogenic variant (Class 5) (Richards, 2015) [4]. The identification of the latter two classes (4 and 5) in *FBN1* establishes the diagnosis of MFS. The detection of VUS (class 3) represents a sort of grey area, and these variants are often challenging for clinicians. VUS interpretation is not static, since its classification can be influenced by multiple ACMG criteria. The diagnostic process must be integrated with different parameters: extensive proband phenotyping, family studies (both genotyping and phenotyping), and possible functional studies. The classification of VUS cases must be re-evaluated over time, since they can be reclassified as likely benign/benign, likely pathogenic/pathogenic, or remain of uncertain significance as they develop.

Fibrillin-1 is an encoded protein composed of 2871 amino acids with an estimated molecular weight of 350,000 Da. This glycoprotein is rich in cysteine and presents in 75% of the length of the 47 tandemly repeating domains of epidermal growth factor (EGF)-like modules (Pereira et al., 1993) [5]. This tandem repetition of EGF-like domains is interrupted by eight cysteine motifs that have homology to a domain known as the TB domain, which was first recognized in transforming growth factor beta (TGFβ) -1-binding protein.

Fibrillin-1 is a ubiquitous protein widely distributed in different tissues and organs including the heart, blood vessel walls, eyes, skin, cartilage, and lungs. Fibrillin-1 monomers form macro aggregates known as microfibrils, which represent an essential structural component in the elastic fibers of the extracellular matrix (ECM). Fibrillin-1 microfibrils play an important role in the extracellular control of TGFβ bioavailability. Its main functions are modulating ligand distribution, storage, and release (Robertson et al., 2015; Asano et al., 2022) [6,7].

**Figure 1:**
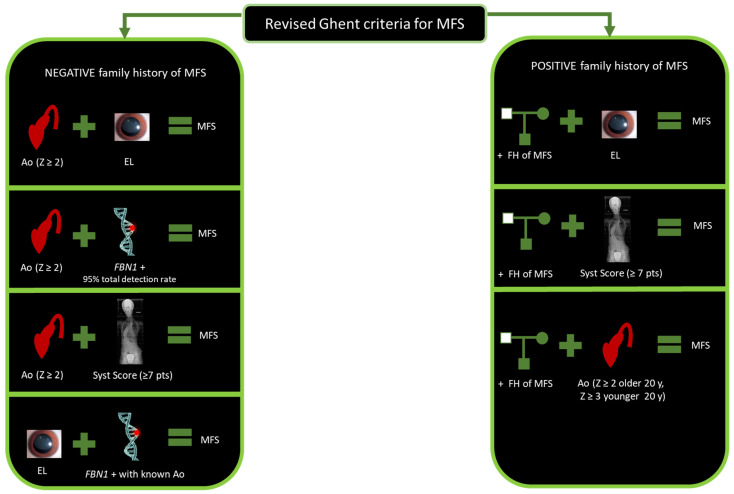
Revised Ghent criteria for MFS diagnosis. Abbreviations: Ao, aorta; EL, *ectopia lentis*; *FBN1* +: *FBN1* positive genetic analysis; FH, family history; y, years; MFS, Marfan syndrome; Syst, systemic; Z, Z-score.

The presence of Fibrillin-1 in various tissues explains the extreme variability observed in clinical manifestations of MFS in terms of involved organs and severity of the disease. International diagnostic criteria are based on Ghent Nosology and were revised in 2010 (Figure 1) (Loeys et al., 2010) [8]. It is known that the two organic structures most involved in MFS are the aorta and the eye. In fact, the two major clinical signs included in diagnostic criteria are aortic root dilatation and *ectopia lentis* (EL).

Aortic root dilatation represents a risk factor for aneurysmatic progressive changes and subsequent rupture. The 2010 revised Ghent Nosology considers aortic changes for the establishment of MFS diagnosis based on two main categories. In the first category, when family history is negative, MFS is confirmed if the aortic root Z-score ≥ 2 in concomitance with EL or *FBN1* variant or a systemic score ≥7 points. In the second category, MFS is confirmed when the aortic root Z-score ≥ 2 if the patient is older than 20 years old, or ≥3 if the patient is under 20 years old, coupled with a positive family history of MFS (Loeys et al., 2010) [8].

EL is defined as a dislocation or displacement of the natural crystalline lens from the patellar fossa. The lens is considered dislocated when it lies completely outside of the hyaloid fossa, it is free floating in the vitreous anterior chamber, or it lies directly on the retina. The lens is defined as being subluxated when it is partially displaced, but remains within the lens space (Kaur and Gurnani 2022) [9].

**Figure 2:**
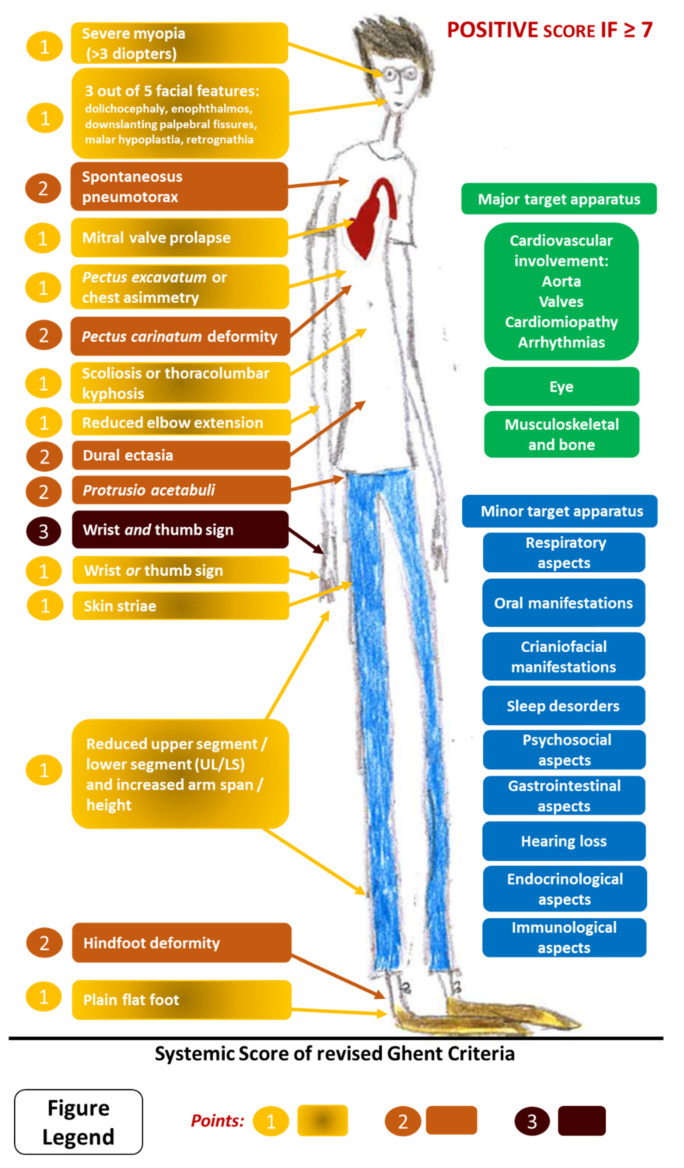
Systemic score of revised Ghent criteria for MFS diagnosis and possible multisystemic involvements in MFS. Please note the three principal aspects involved in MFS are indicated in green: cardiovascular, ocular, and musculoskeletal/bone criteria.

The systemic score is based on the observation of minor clinical changes, such as musculoskeletal features (wrist and thumb signs, *pectus excavatum* or *carinatum*, scoliosis and thoracolumbar kyphosis, reduced elbow extension, plain flat foot, hindfoot deformity, skin striae, *protusio acetabulae*, reduced upper segment/lower segment, and increased arm span/height ratio), facial features (dolichocephaly, downward slanting palpebral fissures, enophthalmos, retrognathia and malar hypoplasia), myopia, mitral valve prolapse (MVP), pneumothorax, and dural ectasia. The systemic score can be considered the third cardinal point in MFS diagnostic criteria (Figure 2).

Application of the revised Ghent criteria can therefore be difficult, especially in younger patients. Thus, in childhood, it is possible that the incidence rate of the disorder is underestimated, or that it is often not diagnosed in a timely manner (Willis et al., 2009) [10].

## 2. Materials and Methods

We performed a review of previous studies documenting clinical manifestations and management in the MFS pediatric population. We searched PubMed for published studies without restriction on the date of publication and without restrictions on language, using the search terms “Marfan Syndrome”, “*FBN1*” AND “children” AND “aorta”, OR “valves”, “arrhythmia”, “cardiomyopathy”, “eye”, “musculoskeletal/bone”, “oral”, “craniofacial”, “hearing loss”, “respiratory”, “sleep disorders”, “gastrointestinal”, “endocrinological”, “psychosocial”.

Review and cohort studies with the following specificity were included: MFS + specific organ and tissue involvement, or clinical and investigational management + pediatric population.

The papers were carefully read and reconsidered according to the abovementioned criteria. Two investigators performed the search independently. The references of the selected papers were crosschecked with the same inclusion conditions. Duplicates were removed.

## 3. Results

From an overview of the literature revision regarding MFS in children, two major distinctive categories can be observed: early onset MFS (eoMFS) (previously known as neonatal MFS, nMFS) and classical childhood onset MFS. Due to the peculiar characteristics of the first category, a separated paragraph has been included to report the major characteristic features related to eoMFS, considering also marfanoid–progeroid–lipodystrophy syndrome (MPLS).

**Figure 3:**
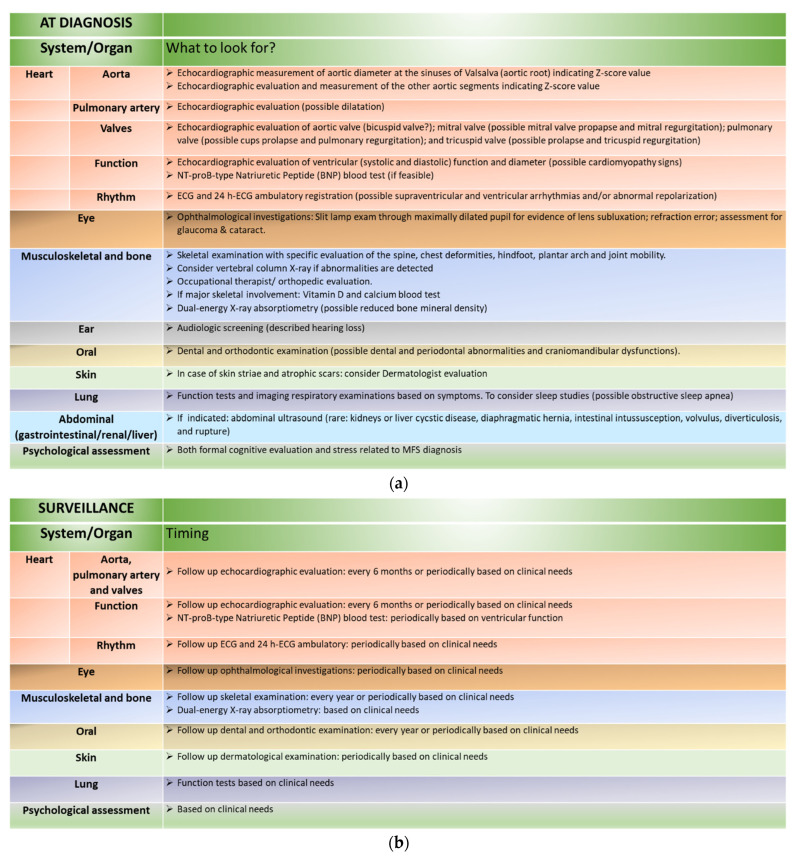
(**a**) Organ/systemic clinical assessment at diagnosis of MFS in children. (**b**) Organ/systemic clinical assessment in the surveillance of MFS in children.

In the subsequent paragraphs, we have included a system-by-system revision for major (cardiovascular, ocular, and musculoskeletal) and minor (recently emerging) characteristic features in children with classical MFS. A systematic approach to organ/systemic clinical assessment at diagnosis and surveillance of MFS children is summarized in Figure 3a,b. Crucial issues in the management and counselling of families with MFS children include the high phenotypic variability and age-related penetrance of the condition, which call for a personalized approach to follow-up programs based on a patient’s health and quality of life.

### 3.1. Early Onset MFS (eoMFS) and Marfanoid–Progeroid–Lipodystrophy Syndrome (MPLS)

EoMFS, previously widely known as nMFS, can be considered a distinctive condition that is typically more severe than classic MFS. The age of onset of eoMFS is not clearly defined, but most of the data included in the literature reports early onset, from the perinatal phase up to the first few years of life. EoMFS is rarer than classical MFS, reported in less than 100 patients in the literature, and is usually caused by *FBN1* (L.) pathogenetic variants located in exons 24–32 (96% of cases) (Zarate 2022) [11].

In this specific MFS population, the family history of MFS is frequently negative (about 62–78% of cases) (Veiga-Fernandez 2020) [12]; indeed, most of the cases are *de novo* in origin for *FBN1* variants (Le Gloan 2016) [13]. The prognosis is often poor, with high mortality within two years of life (mean survival 16.3 months) (Tognato 2019) [14].

Typically, eoMFS patients show extreme musculoskeletal involvement, with conditions such as arachnodactyly (97%), join contractures (87%), pectus deformities, scoliosis, pes planus or thin skin being combined with severe cardiac involvement, which can be diagnosed within a progeria-like spectrum. In this specific category, valvular abnormalities are very common, including MVP (69%), mitral regurgitation (89%), and tricuspid regurgitation (62%), associated to aortic root dilatation in 96% of the cases.

Compared to classic MFS, in eoMFS, mortality is not principally related to aortic dissection or rupture, but to congestive heart failure (CHF), secondary to massive valvular regurgitation with indications for surgical intervention. Prognosis can be also negatively influenced by the presence of congenital emphysema and diaphragmatic hernia (Stheneur, 2011) [15]. Lens dislocation is reported in 64% of the cases.

The need for early diagnosis and dedicated therapeutic and interventional programs represent a critical issue in eoMFS. Recently, Zarate and colleagues proposed a dedicated clinical scoring system that was calculated on the basis of an algorithm that includes the specific features related to the severity and age of onset of the condition. It included nine clinical parameters, and one molecular parameter: four cardiac features (MVP, mitral regurgitation, tricuspid regurgitation, and aortic root dilatation), five systemic parameters (arachnodactyly, join contractures, pulmonary disease, facial features, and lens dislocation), and an *FBN1* variant (variants located within exons 24–32). The combination of clinical parameters (cardiac and systemic). A specific scoring system based on the severity and age of observation of each specific feature lead to the production of a specific cutoff level. The authors described that a cutoff score of ≥14 points showed high sensitivity (100%) and specificity (92%) for establishing the diagnosis. The authors underlined the importance of the clinical score because the identification of a (L.) pathogenic variant in exon region 24–32 is not sufficient to confirm a diagnosis of eoMFS (Zarate 2022) [11].

Another very rare, particular, and early onset phenotype related to *FBN1* variants specifically located in exon 64 occurs in a progeroid disease known as MPLS, which comprises lipodystrophy and partial manifestations of MFS. To date, less than 10 patients have been reported with *de novo FBN1* variants. The clinical spectrum includes intrauterine growth retardation and generalized subcutaneous fat reduction, except in the breast and iliac regions, leading to a senile appearance of the face at birth. From a practical point of view, it is difficult to clinically distinguish eoMFS from MPLS. The clinical features have a high level of overlap with eoMFS, including aortic root dilatation and MVP, EL, joint hypermobility, arachnodactyly, lumbosacral dural ectasia, *pectus excavatum*, and myopia (Passarge 2016; Lin 2020) [16,17].

### 3.2. Classical MFS in Children

#### 3.2.1. Cardiovascular (CV) Involvement

Cardiovascular (CV) manifestations of MFS involve different parameters that must be taken in consideration:

- Patients may exhibit congenital cardiovascular defects (MVP, neonatal dilated aortic root (rare), and associated congenital heart defects (CHDs)). However, CV involvement is often an age related penetrance feature. In other words, progressive aspects can prevail.
- Although CV involvement in MFS is a major component contributing to morbidity and mortality, the CV system can remain intact in a certain proportion of individuals (including adults) with MFS. In other words, CV manifestations can occur in up to 50 to 75% of MFS population. This percentage is under-represented in children due to the potential progressive nature of the disease.
- CV features are not limited to classical aortic dilatation. MFS might be considered a prototype for CV involvement in joint hypermobility disorders. CV changes can be structural (e.g., changes in the bicuspid aortic valve (BAV) and abnormal MVP), progressive (e.g., plurivalvular regurgitation and aortic root dilatation), dysfunctional (hypertension and primary myocardial changes (cardiomyopathy—CMP)), or arrhythmic (second cause of sudden cardiac death—SCD in MFS).

In the selected and eo-forms of the condition, CV involvement is often the leading determinant for MFS diagnosis. Echocardiography represents the gold standard for examining valvular or aortic abnormalities. Aortic root measurements must be interpreted with respect to age and body surface area. A center with an expert pediatric cardiothoracic surgeon might be mandatory when facing CHDs, severe valvular dysfunction, or severe aortic dilatation. Secondary level imaging processes, such as angio-computed tomography (angio-CT) and magnetic resonance angiography (MRA), are frequently performed for point 0 aortic measurements and procedural risk stratification, respectively. The latter investigations can provide more accurate assessments of the cardiac and other vascular (cerebral and abdominal) districts (Baban and Castori, 2018) [18].

In the following sections, major CV manifestations are discussed, with a particular focus on children with MFS.

##### Aorta

The main CV abnormalities in MFS comprise aortic involvement, which represents an important risk factor in case of dissection. Aortic root dilatation is a major clinical criterion in the revised Ghent Nosology. However, it is important to take into consideration two main aspects in counselling for families with children affected by MFS: first, the variable expressivity of aortic involvement (up to 75%) in MFS (Wozniak-Mielczarek, L. et al., 2019) [2,19], and secondly, the age related penetrance of the condition, which is an indication for periodic cardiac evaluations.

It is important to mention the main anatomical landmarks that can help in understanding the red flags that can raise clinical suspicion and ultimately lead to the diagnosis of MFS. The proximal part of the left ventricle (LV) includes the aortic root, which is an ensemble of distinct entities: the annulus (ventriculoaortic junction), the interleaflet tringles, the aortic valve leaflets, the leaflet attachments, the sinuses of Valsalva, and the sinotubular junction. This part is followed superiorly by the ascending aorta, aortic arch, descending thoracic aorta, and abdominal aorta.

Dilatation of the aortic root is mostly symmetrical and limited to the root, especially at the initial stages of the disease. Typically, dilatation is located at the sinus of Valsalva (van Kimmenade et al., 2013) [20]. Therefore, accurate and reproducible measurement of the aortic root diameter is a crucial point in the diagnosis of the condition, as well as the follow-up and planning of medical or surgical management.

In the adult population, it is common to use unadjusted absolute aortic root diameters for clinical and surgical decisions. This aspect is not applicable in the pediatric population due to specific limits related to progressively changing parameters, and for this reason, echocardiographic measurements are expressed in terms of a Z-score, which describes how many standard deviations measurements are above or below a mean value. The parameters that are taken in consideration for such measurements include age, sex, height, and weight (body surface area). The Z-score system can help in identifying patients at risk of developing aortic disease, since only 2.3% of the general population has a Z-score > 2. This proportion is even less (0.13%) when considering a Z-score > 3. In the latest MFS diagnostic criteria, the proposed Z-score for diagnosis in children was set at 3 (Loeys et al., 2010) [8]. A lower threshold was not considered (i.e., >2) since slight differences in measurement can create a relatively major impact on Z-score in children compared to adults, and in clinical practice, moderate aortic dilatation in early childhood can disappear later in life (Gautier et al., 2010) [21]. However, there are no multicentric studies to support a concrete decision as to whether to consider the >2 or >3 threshold at the moment of diagnosis.

Caruana et al. proposed a Z-score threshold governed as well by systemic score. In other words, they suggested the significance of a >3 Z-score to be associated with potential aortic disease. However, this threshold gets down to >2 when associated to major systemic involvement or progressive aortic dilatation. Children in the grey zone (between 2 and 3 Z-score) without clinical manifestations need echocardiographic surveillance every 1–2 years (Caruana et al., 2022) [22].

In 2021 Rutten and his group compared four Z-score calculation methods in children (Pettersen 2008, Gautier 2010, Colan 2006, Lopez 2017) [21,23,24,25], reflecting that different nomograms can lead to clinically important differences in Z-scores, especially in children with a relatively large aortic root diameter (Rutten et al., 2021) [26].

Regarding the measurement method, there is no standardized unique nomogram, and each has its own characteristics and pitfalls. Campens et al. proposed the most updated reference echocardiographic aortic values with the recommendation to use the same method and nomogram for a given patient, especially for follow-up purposes. This recently proposed method includes the use of leading edge to leading edge aortic measurements (Campens et al., 2014) [27]. Another reported Z-score measurement method includes height in patients who are under or overweight (Devereux et al., 2012) [28]. Other tools might be based on multiple calculators, such as those online (online calculation https://www.marfan.fr/accueil/z-score-calculus, accessed on 30 March 2024). One particular measurement, presented by the Marfan Foundation, is based on a study by Colan and colleagues (Colan et al., 2006) [23].

The normal growth rate of the aortic root in unaffected individuals is 0.1 mm/year (Turkbey, E. B. et al., 2014) [29], while it is estimated to be 0.5–0.8 mm/year in children with MFS on medical therapy, although it can reach up to 3 mm/year. This rate is slower in adults (0.3–0.7 mm/year), with the fastest growth observed in men and those with higher root diameter at time of diagnosis. Rates also vary based on treatment regimen (Hascoet, S. et al., 2020; Wozniak-Mielczarek, L. et al., 2019) [19,30].

Ascending tubular aortic dilatation can be observed in MFS, mostly associated to dilatation of the aortic root. This pattern is reversed in patients with BAV aortopathies, in which the tubular ascending aorta is more majorly involved than aortic root dilatation. The largest diameter of the ascending aorta, independently from being the root or tubular ascending aorta, must be applied for determining prophylactic repair (Milewicz et al., 2021) [31].

The presence of BAV in MFS is similar to the general population; in fact, the BAV prevalence ranges from 1% to 2% in the general population and is about 1.8% in the MFS population (Fedak, 2002) [32]. Interestingly, MFS patients with BAV have predominant aortic root involvement and dilatation compared to MFS patients with normally composed aortic valves (Milleron 2019) [33].

Conservative management

Regular cardiac screening and lifestyle modifications are the first step in protecting the aorta. A conservative therapeutic approach should be considered at early stages and periodically reevaluated based on aortic echocardiographic measurements. A proper therapeutic approach can have a considerable impact on natural history and lifespan in MFS by reducing the progression of aortic dilatation. This therapy should be managed by a specialist familiar with its use (usually a pediatric cardiologist or clinical geneticist). The mechanisms of action of implicated molecules, beta-blockers (β-blockers) and angiotensin receptor blockers (ARBs), are mainly related to myocardial inotropy, chronotropy, or targeting signaling pathways involved in MFS animal models. The therapeutic protocol should be initiated at MFS diagnosis at any age, or upon evidence of progression of aortic root dilatation, even in subjects without a definitive diagnosis (Dietz 2001; Baban and Castori 2018) [1,19].

Since the 1990s to date, multiple studies have shown slower aortic root dilatation in patients who were treated with β-blockers and/or ARB compared to those not treated using a pharmacological approach (Shores et al., 1994; Silverman et al., 1995; Erbel, R. et al., 2014) [34,35,36].

**Figure 4:**
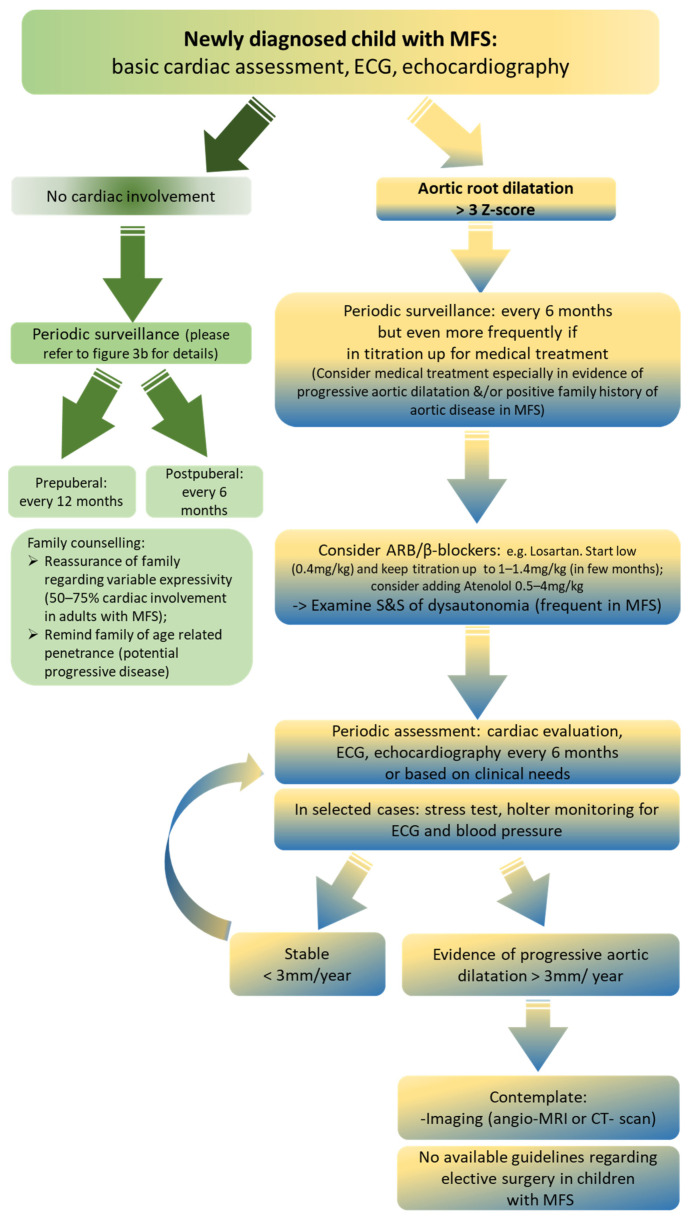
Flowchart for MFS cardiac involvement, adapted from our institution as the regional referral center for children with MFS. Abbreviations: angio-MRI, angio-magnetic resonance imaging; ARB, angiotensin II receptor blockers; CT-scan, computed tomography scan; ECG, electrocardiogram; MFS, Marfan syndrome; S&S, signs and symptoms.

Nowadays, there are no doubts regarding the benefits of medical treatment in reducing aortic root progression in patients with MFS. However, debate remains surrounding the choice of single ARB or β-blocker treatments separately or in combination. Recently, Pitcher et al. performed a meta-analysis of randomized trials of these pharmacological treatments. Studies comparing ARBs versus controls and ARBs versus β-blockers were included, indirectly deriving data about β-blockers versus controls. All included patients were treated before aortic surgery. Results showed that ARBs reduced the rate of aortic dilatation progression in terms of Z-score by about one half; the effects of β-blockers were similar to ARBs. Combination therapy (ARBs and β-blockers), when tolerated, also reduced the rate of enlargement of the aortic root by at least one half, delaying the need for surgery (Pitcher et al.) [37] (Figure 4).

Another issue to be considered in regard to patients with MFS is the indication to continue pharmacotherapy even after surgical procedures, due to the risk of developing distal aortic dissection (Song, H. K. et al., 2012) [38].

Regarding the majority of children with MFS without apparent aortic involvement, especially those diagnosed secondary to positive family history of MFS, determination of appropriate medical treatment can be puzzling. At a final point, after full penetrance of the disease, aortic involvement is not **pathognomonic**, and it occurs in up to 75% of the total adult MFS cohort. Treating *a priori*, for life, a MFS child with normal aortic parameters might not be the best option. The golden rule remains maintenance of echocardiographic surveillance (please see the above paragraph for more details), which is key in detecting potential progressive aortic dilatation and making subsequent decisions regarding potential medical treatments.

Another aspect to be taken in consideration in children with MFS is the impact of physical activity and lifestyle measurements. There are no current sport recommendations for MFS, and knowledge gaps continue to exist. Isometric exercises that require straining or Valsalva involvement should be avoided (Dietz 2001–2022 Genereviews) [1]. Participation in competitive dynamic sports and high-intensity physical activity might be discouraged, particularly in cases presenting a dilated aorta. Frequent ‘burst’ exercises and collision sports are likely risky activities and can favor excessive aortic enlargement and trigger an acute aortic dissection. A personal observation of caution should be taken in consideration when exercising at an intense level, and when playing wind musical instruments that require excessive Valsalva maneuvers. Wind musical instruments, when played at a professional level, might have an exacerbating effect on aortic dilatation mechanisms. However, further studies are needed to confirm this observation.

Conversely, capable individuals with MFS should be encouraged to engage in low-to-moderate aerobic activities, low-intensity recreational sports, and even the use of lightweights (multiple repetitions without straining). Certainly, a degree of caution is appropriate when advising exercise to those with inherited thoracic aortic disease (Cheng et al., Monda et al., Jouini et al., Dietz 2001–2022 Genereviews) [1,39,40,41].

In general, most MFS patients should exercise regularly at a non-strenuous pace, or at about 50% of capacity. It is recommended to avoid contact sports, intense weight training, and isometric exercises, since shear stresses on the aorta might accelerate aortic dilatation. Physical activity, when surveilled by a sports medicine specialist, can lead to improved physical and mental outcomes (Hiratzka, L. F. et al., 2010) [42].

Surgical management of aortic dilatation

Surgical management in children with MFS is an unusual event within pediatric cohorts. Consequently, no multicentric studies have gained consensus status in this very specific field. The age and BSA of the child, the extent of aortic dilatation, preoperative haemodynamic stability, associated arrhythmias, and HF are probably major factors determining outcomes of these procedures (Tirone David, 2019) [43]. Many children with MFS reach “adult” BSA presentation at 10–12 years of age. It is thought that repair of the aortic root and mitral valve can be performed more safely starting at this age. However, the prognosis is usually poor when surgery is indicated during infancy. This is even worse when surgery is indicated on the aorta in neonates.

One of the earliest related studies in the literature was from The Johns Hopkins Hospital and was based on the results of aortic root replacement, mitral valve repair or replacement, and combinations of aortic root and mitral valve operations in 26 children with a mean age of 10 years (range 8 months to 17 years) (Gillinov et al., 1997) [44]. No mortality was reported and overall survival at 10 years was 79%, with 41% freedom from reoperation. suggesting that reoperation was indicated in more than half of the children within a decade of the initial procedure. There have been a few reports on the outcomes of aortic valve sparing operations in children with fairly good results (Fraser et al., 2019; Kluin et al., 2016) [45,46]. Reimplantation of the aortic valve has provided better outcomes than remodeling of the aortic root or composite replacement of the aortic valve and ascending aorta, particularly in children older than 10 years of age.

A multicentric retrospective and prospective registry on surgical management of children with a proven diagnosis of MFS would improve our knowledge and skills to better serve this population. Surgical management of children with MFS should be conducted in specialized centers, given that perioperative complications are frequent and can be unpredictable at a multisystemic level (e.g., respiratory and infective complications and delayed wound healing).

In the adult population with MFS, surgery is indicated with a maximal aortic diameter ≥50 mm. In MFS patients with additional risk factors (see below), surgery should be considered at lower threshold (≥45 mm). Major additional risk factors include: a family history of aortic dissection (or personal history of spontaneous vascular dissection); severe valvular regurgitation (aortic or mitral); fertile women; systemic hypertension; aortic size increase >3 mm/year (on repeated measurements using the same electrocardiogram (ECG) gated imaging technique, measured at the same level of the aorta, with side-by-side comparison, and confirmed by another technique); dyslipidemia; and atrial fibrillation (AF) (Vahanian et al., 2022) [47].

It is important to take into consideration that there are no current guidelines regarding aortic root replacement in children with MFS, although some experts suggest the adaptation of adult aortic roots for children.

A multicentric retrospective and prospective registry on surgical management of children with a proven diagnosis of MFS would improve our knowledge and skills to better serve this population. Surgical management of children with MFS should be managed in specialized centers since perioperative complications are frequent and can be unpredictable at the multisystemic level.

##### Valves

Tricuspid valve

Tricuspid valve anomalies are not considered suggestive of MFS. However, several studies show that frequent tricuspid valve prolapse (TVP) can be detected in MFS. In 2010, Rybczynski and colleagues reported a TVP prevalence of 22% in 204 MFS patients (Rybczynski et al., 2010) [40]. Prevalence appears to be lower in the first two decades of life (4% in 0–10 years and 17% in 10–20 years), increasing in age groups up to 40 years (29% in 20–30 years and 32% in 30–40 years). In a 2020 study published by Bombardieri et al., observed a slightly lower TVP prevalence (13%) in a cohort of 69 *FBN1* positive patients with a median age of 23 years (range 1–75) (Bombardieri et al., 2020) [48].

An interesting finding by Rybczynski and his group was that TVP was commonly associated to MVP with a severe grade of regurgitation (Rybczynski et al., 2010) [40]. Concurrent involvement of the mitral and tricuspid valves was reported by Gu in 9.6% of MFS adults (seven patients of 73) (Gu et al., 2015) [49].

Limited data are available concerning tricuspid valve disease in MFS children. A study on a small pediatric cohort (seven patients) showed a TVP prevalence of 63.6% (Ozdemir et al., 2011) [50]. Recently, Stark and his collaborators performed a retrospective and observational study focused on TVP and its predictive value for the outcome of pediatric MFS. They found TVP in 68.5% of patients (89/130 aged 10.7 ± 4.8 years). TVP seems to be a possible marker of potentially more aggressive cardiovascular and systemic phenotypes; in fact, at baseline, children with TVP had a higher incidence rate of aortic root dilatation, MVP, and systemic manifestations than patients without TVP. Also, at follow-up, aortic root dilatation and systemic manifestations were more present in the TVP group than the non-TVP group. Considering that TVP appears early in the disease course, the authors concluded that TVP should be routinely and regularly assessed in the echocardiography of MFS children as a possible predictor of clinical severity outcome (Stark et al., 2022) [51].

There is no standard protocol for TVP and related regurgitation. The measurement of the entity of this parameter is evaluated as part of the echocardiogram surveillance conducted in MFS. In cases of severe regurgitation or symptomatic patients with right-sided HF, surgery might be indicated (Vahanian, 2022) [47].

##### Mitral Valve

Mitral valve disease represents the other major cardiac involvement in MFS beyond aortic root dilatation. MVP is one of the systemic MFS score criteria. In MFS adults, the prevalence of MVP is higher compared to the general population (40–68% versus 1–2%) (Milewicz et al., 2021; Pyretz et al., 1993; Muhlstadt et al., 2019) [31,52,53]. MVP is also a relevant manifestation in the pediatric MFS population, occurring in 32% to 38% of cases, and the prevalence is age-dependent (Mueller et al., 2013; Selamet Tierney et al., 2013) [54,55].

In 2010, Rybczynski and colleagues performed a large population-based cohort study focused on mitral valve dysfunction in MFS, reporting an MVP cumulative distribution of 8.7% at 1 year of age, 26.7% at 10 years, 36.1% at 20 years, 42.6% at 30 years, 47.7% at 40 years, 51.8% at 50 years, and 60.8% at 80 years of age. Severe mitral valve regurgitation increases with age: the estimated cumulative distribution is 0 at 1 year, 2.5% at 20 years, 13.3% at 40 years, 32.7% at 60 years, and 55.8% at 80 years of age. In addition, the same authors found that MFS patients with MVP have a 28% increased risk of complications, such as operations, endocarditis, or HF, compared with 13% in patients with idiopathic MVP (Rybczynski et al., 2010 and Rybczynski et al., 2011) [56,57].

Interestingly, the prevalence of MVP in MFS children and young adults is also gender-related; in fact, Lacro et al. reported that the prevalence of measurable (mild or worse) mitral regurgitation (MR) was higher among female subjects (MVP: females 45% versus males 33%, MR: females 25% versus males 13%) (Lacro et al., 2013) [58].

##### Cardiomyopathy (CMP)

Cardiac involvement in MFS is known to be classically associated with aortic or valvular abnormalities. However, the presence of myocardial changes predisposing to CMP is increasingly emerging in the literature. In the 1950s, the definition of “arachnodactyly heart” was introduced to underline the peculiar myocardial aspects of MFS (Glesby, 1989; Bradley et al., 2016; Fusco et al., 2022) [59,60,61]. Nevertheless, myocardial impairment is frequently related to valvular anomalies and consequent ventricular volume overload. However, it is not possible to exclude *a priori* a “primum” myocardial changes independently from hemodynamic factors. In 1994, Savolainen and colleagues reported that LV early diastolic function is impaired in children with MFS, probably due to primitive connective tissue abnormalities (Savolainen et al., 1994) [62].

Interestingly, in 2021, Mosquera and De Backer defined two possible myocardial expression entities in MFS: HF in neonatal (eoMFS)/infantile MFS, and CMP in classical MFS. As discussed in Section 3.1, in the eoMFS population, it is common to observe hemodynamic abnormalities related to valvular prolapse (tricuspid/mitral), causing severe progressive regurgitation and subsequent CHF. In contrast with classical MFS, aortic root dilatation in eoMFS does not represent the major morbidity factor in this population. In fact, prognosis and survival are mainly related to the severity of valvular involvement and HF symptoms (Mosquera and De Backer 2021, Faivre 2009) [63,64].

Regarding CMP in classical MFS, which can also be defined as “Marfan CMP”, the reported prevalence in the literature is widely variable, ranging from 3% to 68% (Hetzer 2016, Yetman 2003) [65,66]. In Marfan CMP. myocardial impairment can be intrinsic with biventricular involvement, and is associated to both systolic and diastolic dysfunctions (De Backer 2006) [67]. The latter finding seems not to be related to reduced aortic elasticity; in fact, there is no significant relation between LV ejection fraction and flow wave velocity (FWV), a parameter related to aortic elasticity (de Witte, 2011) [68]. This evidence suggests that Fibrillin-1 anomalies are probably causative not only for aortic wall injuries, but also for myocardial changes.

##### Arrhythmias

Structural CV involvement in MFS is traditionally described in the literature. Growing evidence in the last decades has shed light on associated arrhythmic spectrum in MFS. Limited data are available regarding heart rhythm abnormalities in the natural history of MFS in children.

These changes can be classified into three main groups: supraventricular arrhythmias, ventricular arrhythmias, and repolarization disorders. Supraventricular (SV) and ventricular arrhythmias have been reported in the literature in MFS populations (both children and adults), independently from valvular involvement (Aydin et al., 2013; von Kodolitsch et al., 2019) [69,70].

Three studies have reported life-threatening arrhythmias in 7–9% of MFS patients, along with SCD, most likely due to arrhythmia, in up to 4% (Yetman et al., 2003, Aydin et al., 2013) [66,69]. Major causes of cardiac death include aortic dissection/rupture, HF, and ventricular arrhythmias (Gott et al., 1999; Yetman et al., 2003) [66,71].

Attempts to achieve arrhythmic risk stratification in MFS reported some involved parameters: ventricular ectopy (VE), non-sustained ventricular tachycardia, ventricular tachycardia, LV systolic dysfunction, and elevated serum N-terminal pro-brain natriuretic peptide levels (NT-proBNP) (Hoffmann et al., 2013) [65]. The latter seems to be the strongest independent predictor of arrhythmogenic events (Aydin et al., 2013, Hoffmann et al., 2013) [69,72].

Supraventricular (SV) and ventricular arrhythmias

In children with MFS, SV and ventricular arrhythmias might be underestimated aspects with potential dynamic implications for CV outcome. No serial multicentric studies on pediatric populations with MFS are available.

It is important to take in consideration that VE is classically considered to be a common finding among children, especially when associated with negative familial history, asymptomatic and monomorphic presentation, a common ECG pattern, and it is often suppressed by exercise (Cicenia et al., 2021) [73].

One of the few multicentric studies in the area was conducted in 2018 by Mah and colleagues on 274 MFS children and young adults (11 ± 6 years) with 24 h ECG ambulatory data available for review. Only 7% of patients presented significant VE, and 5% had significant supraventricular ectopy (SVE); two patients (1%) had both. Only two from those with VE had a burden >10% (about 20%). Ventricular tachycardia (VT) or supraventricular tachycardia (SVT) were not reported. On one hand, no correlation was observed among VE and MVP, MR, LV size and function, and aortic dimensions or stiffness. On the other hand, the authors detected a relationship between SVE and dilatation of the sinotubular junction and the aortic stiffness measures. A novel observation from that study included the correlation between a higher heart rate variability (HRV) and increased risk of aortic dissection. It was hypothesized that the greater oscillation in heart rate and increased sympathetic tone may induce higher aortic wall stress, increasing the risk of dilatation and dissection (Mah et al., 2018) [74].

In a single center report from Yetman and his group, 21% of children with MFS presented VE and 6% presented non-sustained ventricular tachycardia. In contrast with the Mah study, a greater prevalence of MVP with MR and LV dilatation was observed with VE (Yetman et al., 2003) [66].

In 2019, a study performed by Wozniak-Mielczarek and collaborators showed no major differences among adults and children with MFS in terms of VE, with a prevalence of 12.28% in adults and 4.55% in children. SVE was detected in five patients with MFS (4.95%), with similar observed prevalence in adults and children, three (5.26%) versus two (4.55%), respectively (Wozniak-Mielczarek et al., 2019) [18].

Recently, Demolder and colleagues performed a large international multicenter retrospective study on a cohort of thoracic aortic disease patients (12 years or older). On one hand, they reported that atrial arrhythmia (atrial fibrillation/atrial flutter) is most common in patients with (L.) pathogenic variants affecting *FBN1* compared to patients with (L.) pathogenic variants in other TGF-β signaling genes. On the other hand, no significant difference was found between these two populations regarding ventricular arrhythmia. They also reported an association between mitral annular disjunction (MAD) and ventricular arrhythmias in MFS patients (Demolder et al.) [75]. Further studies are needed to show the prevalence of these events in the pediatric MFS population.

Repolarization disorders

In a similar manner, two MFS cohorts showed a higher frequency of QTc prolongation >440 ms, reaching up to 9–20% of children with MFS and 16–20% of adults (Yetman et al., 2003 and Savolainen et al., 1997) [66]. QT interval prolongation was unrelated to aortic root dilatation, LV function, or dimension and mitral valve prolapse (Savolainen et al., 1997) [76].

Arrhythmic predisposition, combined with abnormal repolarization, remarkably suggests a subclinical aberration background in the electrophysiological substrate. To date, the mechanism underlying these disorders remains unknown. Based on these observations, careful consideration should be undertaken when prescribing drugs with proarrhythmogenic/QT prolonging aspects.

#### 3.2.2. Eye

Ocular involvement in MFS is one of the major defining criteria, in addition to CV and musculoskeletal involvement. It is imperative to undertake all eye studies in the diagnostic setting of MFS. On one hand, ocular implications and management protocols in MFS adults are well-described in the literature. On the other hand, few studies are focused on the pediatric MFS population.

EL represents a distinctive and severe manifestation in the revised Ghent Nosology, with prevalence reaching ~60% in adult MFS. To the best of our knowledge, no multicentric or large single center studies are available regarding the exact prevalence of this feature in the pediatric MFS population.

In differential diagnosis, EL is not pathognomonic for MFS, and might be associated to ocular trauma, infection, inflammation and tumors (Cross and Jensen 1973; Hayward and Brock 1997; Kainulainen 1994) [77,78,79]. EL is a possible sign of classical homocystinuria (occurring in about 85% of cases). Additionally, isolated EL is also associated to (L.) variants in *FBN1* in dominant traits, or to (L.) variants in *ADAMTSL4* in recessive traits.

In a single center study combining ophthalmic and cardiac department observations, Salchow and Gehle performed a retrospective comparative study on MFS in patients younger than 17 years old (52 children with MFS versus 73 controls). EL was diagnosed in 25/52 (49%) of MFS patients; it was bilateral in 68% of these cases. Age and gender were not significantly different among patients and controls. The most frequent direction of lens subluxation was superior (47.6%). As in adults, EL is frequently associated with a decrease in visual acuity (VA); in fact, EL can cause myopia, irregular astigmatism, and high hyperopia (Salchow and Gehle 2019) [80].

Other common ocular manifestations of MFS are myopia ≥3 diopters (>50%), transillumination defects with increased axial length (AL) (>23.5 mm), flat cornea (<41.5 D average corneal curvature), decreased corneal curvature, and greater corneal astigmatism. In addition to EL, other major ocular complications include retinal detachment (RD) (<25%) and glaucoma (~30%) (Gehle et al., 2017; Dietz GeneReviews 2001–2022) [1,81].

In adults, MFS eyes have been found to be longer than those of control groups. Based on Salchow and Gehle, this was not the case in children, where the eyes were shorter. Moreover, AL was comparable in children with MFS with and without EL, which contrasts with reported data in adults, in whom EL is associated with increased AL. This parameter might represent a dynamic aspect of the condition which progressively changes through adolescence (Salchow and Gehle, 2019) [80].

Another ocular characteristic in MFS adult patients is reduced corneal curvature (Gehle et al., 2017) [81]. Salchow and Gehle demonstrated that children with MFS have a significantly reduced average corneal curvature (Kmed) compared with controls, suggesting this parameter as a diagnostic criterion for MFS at pediatric age (Salchow and Gehle, 2019) [80]. In a similar manner, corneal astigmatism in MFS children was significantly higher than in controls, and often associated with EL (Kinori et al., 2017; Konradsen et al., 2012) [82,83].

Kinori and colleagues suggested that corneal parameters should be well established if MFS is suspected, especially in children. This aspect might represent part of a risk stratification tool for those at risk of developing EL.

In Ghent revised criteria, the minor diagnostic criteria of myopia >3 diopter (D) may not be present in children with MFS due to the compensatory effects of a flattened cornea, which balances the increased AL in terms of refractive error. A few studies have reported certain specificities of myopia in children with MFS, where it was detected at a level of >3 D in 34.9% and >0.75 D in 41.9% of MFS children versus 12.9% for both D measurements in the control group (Kinori et al., 2017; Salchow and Gehle 2019) [80,82]. The presence of severe myopia should be considered a red flag for EL.

Another less characterized criterion includes a decreased central corneal thickness (CCT), which can represent a risk factor for primary open-angle glaucoma. In an analyzed MFS cohort studied by Salchow and Ghele, only one patient had suspected glaucoma (Salchow and Gehle 2019) [79]. Lower intraocular pressure (IOP) has been previously reported in children and adults with MFS overall and in association with EL (Drolsum et al., 2015) [84].

Iris transillumination defects (ITD) were diagnosed in a lighter way in MFS children’s eyes (19.6% versus 4.3% in controls). Regarding RD, it was not reported in MFS children, while it is typically observed in adults with MFS. However, early screening for RD risk factors is recommended, with periodic ocular fundus examination (Salchow and Gehle 2019) [80].

Based on the relevant and often severe ocular involvement, it is essential to perform all ophthalmological investigations when MFS is suspected. Especially in children, screening must be early and periodic, at the time of diagnosis, and at least annually or based on clinical needs, because many signs may not initially be present, such as EL or myopia. In addition to standard screening, measurement of AL and corneal curvature should be considered and are obtainable in a clinical setting, even in young children (Kinori et al., 2017) [82].

#### 3.2.3. Musculoskeletal and Bone

Osteoarticular anomalies are extremely common in MFS, and represent the third major feature in this condition, in addition to those observed in the CV and ocular systems. Skeletal changes may involve any segment, but primarily the abnormalities are observed in the chest (*pectus excavatum* or *carinatum*), spine (scoliosis and thoracolumbar kyphosis), and feet (plain flat foot and hindfoot deformity). Other possible features include reduced elbow extension, *protusio acetabulae*, reduced upper segment/lower segment, and increased arm span/height ratio. There are few studies in the literature regarding musculoskeletal aspects in the MFS pediatric population. In 2014, Stheneur and his group performed a large study on 259 MFS children compared with 474 non-MFS children in which they attempted to clarify phenotype evolution during childhood. They interestingly found that skeletal features are very heterogeneous and evolve during growth with an age-dependent mechanism. In detail, the prevalence of pectus abnormalities increased from 43% at 0–6 years to 62% at 15–17 years, wrist signs increased from 28 to 67%, and scoliosis increased from 16 to 59%. Hypermobility decreased from 67 to 47% and pes planus decreased from 73 to 65% (Stheneur et al., 2014) [85].

Recently, an Italian group investigated the correlation between musculoskeletal features and CV abnormalities in 72 MFS pediatric patients (range 3 to 14 years) (De Maio et al., 2021, De Maio et al., 2016) [86,87], showing a statistically significant correlation between *pectus excavatum* and aortic root dilatation with Z-score ≥3.

Another multicentric retrospective study evaluated the correlation between spinal and chest anomalies and pulmonary function in MFS pediatric patients with clinical history of surgically managed spinal deformity. The authors demonstrated an impaired pulmonary function test in 70% of these patients. Kyphosis, chest wall deformity, and increasing coronal curve magnitude are the three most relevant factors related to decreased pulmonary function (Otremski et al., 2020) [88].

Certainly, tall stature can be the leading sign for MFS diagnosis in children, although height is not considered a specific diagnostic criterion. In fact, height can be influenced by many factors, such as target family final height. Initially, some studies proposed specific growth charts for MFS children (Pyeritz et al., Erkula et al., Kwun et al.) [89,90,91]. In 2017, the French research group lead by Benoist developed the first European growth charts (height, weight, bode max index -BMI, and arm span) for MFS children (males and females) on the basis of an observational study of 259 MFS children and 474 variant-negative sibling controls. MFS children (both genders) were substantially taller than control group children at all ages. However, MFS children’s overgrowth reduced with age. At 17 years old, the mean height was 191.2 ± 8.4 cm in the MFS male group versus 182.9 ± 8.1 in the male control group, and 178.3 ± 7.6 cm in the MFS female group versus 169.5 ± 6.8 in the female control group. In contrast, MFS children showed BMI similar to non-MFS children, and inferior to the values observed in the French general population (Benoist et al., 2017) [92].

In addition to the morphological skeletal changes, the state of reduced bone mineralization related possibly to osteoporosis is a potential underestimated indicator in MFS children that has received growing interest in the literature. Most of the studies related to this aspect of the condition have recommended periodic screening of bone mineral status and to evaluate diet, physical exercise, vitamin D status, and bone turnover markers to prevent reduced bone mass (Grover et al., 2012, Haine et al., 2015, Trifirò et al., 2015, Trifirò et al., 2020) [93,94,95,96].

#### 3.2.4. Systemic Manifestations

 Other systemic manifestations are described in Supplementary Materials [97,98,99,100,101,102,103,104,105,106,107,108,109,110,111,112,113,114,115,116,117,118,119,120,121,122,123,124,125,126,127,128,129,130,131,132,133,134].

## 4. Conclusions

MFS is an extremely complex and variable condition. Major organ involvement is not constant. Age penetrance is an important factor to be considered in children with MFS. Initial family counselling must be shared and acknowledged by families. Specific organ surveillance is a fundamental issue, and clinical approaches must be focused on the cardiac, ocular, and skeletal aspects of the condition (Figure 3a,b). Other less frequently reported or underestimated issues must be included at point zero diagnosis in order to recognize dedicated organ/systemic involvement. Overall, in MFS children, a multispecialty management strategy should be the main point of consideration both in the diagnosis and long term follow-up of the condition.

## Data Availability

Details regarding where data supporting reported results is available upon request.

## Supplementary Materials

The following supporting information can be downloaded at: https://www.mdpi.com/article/10.3390/jcdd11040114/s1. The supplementary file includes detailed description of other systemic manifestations that can be of impact on the health of children with MFS. References [97,98,99,100,101,102,103,104,105,106,107,108,109,110,111,112,113,114,115,116,117,118,119,120,121,122,123,124,125,126,127,128,129,130,131,132,133,134] are cited in the Supplementary Materials.
