# Supplementary material for: Unique Features of Cardiovascular Involvement and Progression in Children with Marfan Syndrome Justify Dedicated Multidisciplinary Care
Source: J Cardiovasc Dev Dis. 2024 Apr 3;11(4):114. doi: 10.3390/jcdd11040114 (PMC11050181; doi:10.3390/jcdd11040114)
Supplement: Supplementary file 1 [file jcdd-11-00114-s001.zip › jcdd-2920737-supplementary.pdf]

SUPPLEMENTARY MATERIALS:

Other systemic manifestations of Marfan Syndrome in Children:

Oral manifestations

Oral manifestations in MFS play an important role in the clinical practice. This is mainly divided into three main aspects: 1. higher risk of endocarditis with underlying cardiovascular involvement; 2. functional abnormalities related to malocclusion and periodontal conditions; 3. esthetic aspect related to dental crowding and mandibular abnormalities. The population of MFS occupy a special position in dental practice.

Dental manifestations in MFS are extremely variable including oligodontia or hypodontia, periodontal ligament dysfunction, vulnerability to an increase inflammatory response particularly on periodontal tissue. Patients with MFS are also more likely to have crowded teeth than those without MFS; consequently, oral hygiene care can be more difficult, increasing inflammatory risk (Alam et 2022) [97].

The orthodontic aspect is crucial in the management MFS children due to its functional and esthetic impact. However dental caries is not demonstrated to be at higher percentage is children MFS population (Rahman et al. 2021) [98]. Oral health care must be considered within a program of endocarditis prophylaxis (von Kodolitsch et al. 2019) [70].

In 2019, Venza and colleagues analyzed periodontal clinical parameters in 16 children with MFS in relation to gingival inflammatory condition, showing a higher and early onset Plaque Index (PI) (59% vs 21% in control group) and Bleeding on Probing (BOP) (36% vs 16 % in control group). (Venza et al, 2019) [99].

As part of standard care, children with MFS might need periodic surveillance by dentists familiar with the specific manifestations that can be identified in this specific condition.

Craniofacial manifestations

Craniofacial anomalies in MFS are common; according to Johnson et al retrognathia of le lower jaw and downslanting palpebral fissures are two most prevalent features in MFS population regarding this focus. In addition, MFS patients show a subnormal/abnormally narrow facial width probably related to malar hypoplasia (Johnson et al 2019) [100]. Other frequent aspects include dolichocephaly, high arched palate, a skeletal class II, temporomandibular joint abnormalities including instability. Probably these morphological anomalies can be explained by a different growth of complex and the palatal vault in MFS compared to the general population. (De Coster et al, 2004) [101].

In 2013, Docimo and colleagues examining an MFS pediatric MFS found that 56% of patients had a cross bite (mono- or bilateral) and 69% had a high arched palate. The authors also estimated that the prevalence of cross bite in MFS was 2.5 to 7 times more frequent than the no MFS population (Docimo et el) [102].

Craniofacial manifestations in children with MFS might need to be managed at multidisciplinary level by dentists, craniofacial surgeons, occupational therapists and even ENT specialists.

Hearing

As part of multisystemic evaluation, hearing can be a determinant factor in MFS patients' cognitive and speech development. Systematic research of literature has shown limited results. Hearing loss (HL) is thought to be more prevalent in MFS probably secondary to chronic otitis media, Eustachian tube dysfunction, skull and facial bones anomalies, scoliosis, joint abnormalities, and hypertension.

A recent single center study reported hearing loss in 52.8% within a total cohort of 70 children and young adults with MFS probably correlated to hypertension and linked to microvascular insufficiency causing endothelial dysfunction, leading to cochlear impairment The authors suggested audiologic screening in MFS. (Hamberis et al., 2020) [103]. Further multicentric studies need to be elaborated prior to derive specific conclusions in this subgroup.

Respiratory aspects

Although lung involvement is not considered within the major criteria of MFS, many patients have a variable degree of underlying pulmonary pathology. We classified this aspect into the following three main parts.

Spontaneous pneumothorax (SP)

Several reports describe the association of MFS and spontaneous pneumothorax (SP) among the minor criteria for the clinical diagnosis of MFS (Figure 2). A patient with MFS is probably several hundred times more likely to sustain SP (wood JR et al., 1984) [104]. In the paediatric population, SP is a relatively rare event with a bimodal peak age of occurrence: mostly in the neonatal period (2.2%) or in late adolescence or adulthood (11.9%) (Carolina Viveiro et al., 2013) (Mary J Roman et al., 2017) [105,106].

The increased risk of SP can be attributed to the presence of apical blebs, bullae, abnormal connective tissue constituents in the lung parenchyma or increased mechanical stresses in the lung apices due to the tall body habitus (Karpman C 2011) [107]. Strategic lines for the management of SP in children with MFS depends on several factors: haemodynamic instability, underlying cardiac / aortic involvement, age (eoMFS or paediatric MFS), underlying interstitial lung disease, associated thoracic cage abnormalities, and prolonged signs and symptoms (O'Lone et al, 2008) [108].

Pulmonary changes not specifically related to SP

The pulmonary changes include widespread or patchy cystic changes, emphysema, focal pneumonia or bronchiectasis, bullae, congenital pulmonary malformations (particularly middle lobe hypoplasia), and apical fibrosis (Dyhdalo et al., 2011) (pek Chuan Teoh 1977) (Tun et al, 2021) [109,110,111]. This might be re-lated to the considerable expression of FBN1 in pulmonary tissue (Gene cards: https://www.genecards.org/cgibin/carddisp.pl?gene=FBN1) [112].

As for asthma, wheezing, and reactive airways disease, few studies showed discordant results regarding increased association of this sign in children with MFS (Konig et al. 1991) [113].

Tracheobronchomalacia

Tracheomalacia results in narrowing of the tracheal lumen to less than half by expiratory compression caused by a thin or weak tracheal wall. Sporadic case reports described the association of tracheo / bronchomalacia in young

patients with MFS especially in the neonatal form. Other emphasized this risk during intubation / difficulty in postoperative extubation. Another mechanical aspect that can be observed in eoMFS is the eventration of the hemidiaphragm and main stem bronchomalacia as other atypical and rare pulmonary manifestations previously outlined in eoMFS (Jacobs et al, 2002, Revencu et al, 2004, Young Oh et al., 2002, Shinawi M 2005) [114,115,116,117]. These aspects are of particular importance to intensivist who can use specific precaution methods in respiratory management of this group of patients and to consider them within a category of higher risk individuals with need of personalized medicine approach.

Sleep disorders

The prevalence of obstructive sleep apnea (OSA) is remarkably increased in MFS adults 30-80% (Rybczynski et al. 2010 B) [118], compared to about 10% in the general population (Lurie et al. 2011) [119]. OSA can lead to subsequent progressive and important cardiovascular risk factors, particularly for aortic root dilatation and dissection.

Few studies are present in literature concerning OSA in the pediatric population. In a single limited study of MFS children, OSA prevalence was 80%, compared to 1-5% in the general pediatric population. OSA in MFS children is multifactorial: musculoskeletal abnormalities (pectus excavatum or carinatum, tight chest, and scoliosis), craniofacial alterations (high-arched palate, retrognathia, maxillary constriction and malocclusion) and increased laxity of the soft tissue and upper airway (Paoloni et al, 2008; Marcus et al. 2012) [120,121]. Pulmonary function test can be detected by spirometry exam that can be applied in collaborative patients (>6years old). It can represent a reasonable starting point when respiratory disorders are suspected especially in patients with narrow AP chest diameter and progressive functional abnormalities at vertebral level (severe progressive scoliosis).

In 2021 MacKintosh and colleagues investigated the usefulness of non-invasive OSA screening tools in children with MFS through retrospective polysomnogram (PSG), Pediatric Sleep Questionnaire (SRBD-PSQ) and Epworth Sleepiness Scale (ESS-CHAD) scores. The data collected showed that standard screening questionnaires for OSA may be inadequate in MFS children suggesting that this screening modality may not reliably identify patients at risk in this population. For this reason, the authors suggested systematic and regular polysomnography in pediatric MFS, even in the absence of classical symptoms of sleep apnea. (MacKintosh et al, 2021) [122].

The early identification and appropriate management of MFS children with OSA can lead to an improvement in aortic prognosis, reducing cardiovascular morbidity and mortality. Moreover, OSA management can have impact on sleep disorders, growth, behavior, and cognitive consequences (poor school performance, inattention, attention-deficit/hyperactivity disorder-ADHD, daytime fatigue, insomnia).

Gastrointestinal aspects

Some case reports of gastrointestinal complications of MFS in pediatric patients are reported in literature so far: three eoMFS cases with intrathoracic stomach and one eoMFS case with intrathoracic gastric volvulus surgically corrected, a case of spontaneous rupture of the colon sigmoid following spontaneous recurrent pneumothorax in a 17-year patient and a delayed presentation of Morgagni hernia, a type of congenital diaphragmatic hernia in a 9-year girl. (Petersons et al, 2003) , (Herman et al, 2013) , (Kourinis et al, 2006) , (Ayse Esin et al, 2010) [123,124, 125,126].

Recently, Maconi and his group described 90 MFS patients (age 7 to 70 years). In that study children did not show a greater prevalence of diverticula or a statistically significant sonographic thickening of the muscularis propria compared to controls or adults with MFS (Maconi et al., 2020) [127].

Endocrinological aspects

Endocrinological aspects of pediatric MFS are not well characterized so far. Until now, there are few case reports of pediatric MFS patients with diabetes mellitus (Yamamoto et al., 1992) (Zhou et al., 2019) [111,128,129].

Recently, some authors described mouse models of MFS focusing on the association between adipose tissue depletion and MFS lipodystrophy phenotype, providing a thorough characterization of metabolism and bone remodeling in mouse models of MFS (Davis et al., 2016) (Walji et al., 2016) [130,131].

Immunological aspects

As for endocrine system, immunological diseases of MFS pediatric patients are not well characterized until now. A higher prevalence is observed of eosinophilic esophagitis in inherited CTDs (Abonia et al., 2013) [132]. However, in this study there was not specific description of the association of eosinophilic esophagitis to which category of CTDs.

Psychosocial aspects

The management of the psychological spectrum in MFS children is crucial in the growth process and in consideration of the future adult life. Indeed, one of the most underestimated clinical aspect in MFS children is the psychosocial consequences of growing up with this condition. Up to date, very few data in literature have studied whether MFS children do have impaired Health-Related Quality of Life (HRQoL) and mental health. Recently a study by CTD study group compared HRQoL and mental health among the majorsubgroups including MFS. The study concluded that children with MFS showed decreased HRQoL but not for mental health (Warnink-Kavelaars et al., 2021) [133].

It isimportant to remember that children with MFS may face the practical difficulty of "appearing" older than their age due to tall stature or certain dystrophic aspects. These factors can lead to "false" expectations of cognitive or mental performance from lay people (teachers, coaches). It might be of help to teach these figures to remember the actual age rather than the "size" age prior to giving duties to children with MFS.

Other less frequently noticed conditions with psychological impact in MFS include different forms of pain with localized and acute (nociceptive) pain associated with episodic joint microtraumas. Dislocations are rare in children with MFS but need appropriate management (Baban and Castori 2018) [19].

In a recent study, van Andel and colleagues demonstrated that MFS adult population show severe fatigue compared to general population over all in females. In addition, on one hand, older MFS patients with severe cardiovascular impact andchronic pain show subsequently major anxiety symptoms. On the other hand, unemployment adult MFS patients present depression compared to younger MFS patients or to general population. The authors stress the importance of psychological assessment and intervention, as cognitive behavioral therapy, to reduce the possible impact of psychological symptoms in term of better quality of life. (van Andel 2022) [134].
